# Insights from the bottom-up development of LGR5-targeting immunotherapeutics

**DOI:** 10.1093/immadv/ltaf017

**Published:** 2025-04-25

**Authors:** Nico Mueller, Marc Andrew de la Roche, Maike de la Roche

**Affiliations:** Cancer Research UK Cambridge Institute, University of Cambridge, Cambridge CB2 0RE, United Kingdom; Department of Biochemistry, University of Cambridge, Cambridge CB2 1GA, United Kingdom; Cancer Research UK Cambridge Institute, University of Cambridge, Cambridge CB2 0RE, United Kingdom

**Keywords:** LGR5, immunotherapy, CRC, HCC, pre-B ALL, ADC, bispecific engager, CAR T cells

## Abstract

Leucine-rich repeat-containing G-protein coupled receptor 5 (LGR5), a transcriptional target gene of the Wnt signalling pathway, is overexpressed in multiple cancers, including colorectal cancer (CRC), hepatocellular carcinoma (HCC) and pre-B acute lymphoblastic leukaemia (pre-B ALL) and has emerged as a promising therapeutic target. Here, we reflect on the bottom-up development of a novel α-LGR5 therapeutic antibody we have recently reported, into a palette of LGR5-targeting immunotherapeutic modalities: antibody-drug conjugates (ADCs), bispecific T cell engagers (bispecific engagers), and chimeric antigen receptor (CAR) T cells. The α-LGR5 antibody is highly specific and accurately detects LGR5 protein expression levels, enabling its use as a prognostic biomarker for identifying LGR5^+^ tumour types. Preclinical studies road-testing the various α-LGR5-based modalities established potent and safe elimination of LGR5-expressing cancer cells *in vitro* and efficacy in a mouse model of human cancer *in vivo*. In this review, we discuss the utility of our antibody as the building block for a novel set of immunotherapeutics and highlight the importance of matching specific α-LGR5-based therapeutic modalities to individual tumour type and patient characteristics.

## Introduction

We have recently reported the development of α-LGR5, a unique monoclonal antibody raised against the stem cell marker Leucine-rich repeat-containing G-protein coupled receptor 5 (LGR5). α-LGR5 fills the need for an effective reagent to detect low LGR5 expression in certain stem cell compartments and overexpression in cancer cells. Our substantial validation studies establish α-LGR5 as an excellent research tool for detecting LGR5 protein expression. From this starting point, we have carried out immunotherapeutic development of α-LGR5 in the ADC, CAR, and bispecific modalities in an academic research programme that we refer to as “bottom-up therapeutic development”. Here we describe α-LGR5’s remarkable versatility that enables matching of properties of different immunotherapeutic modalities to cancer- and patient-specific characteristics in order to optimize the effectiveness of cancer treatment.

## LGR5 is an attractive target for immunotherapies

**Figure 1.**
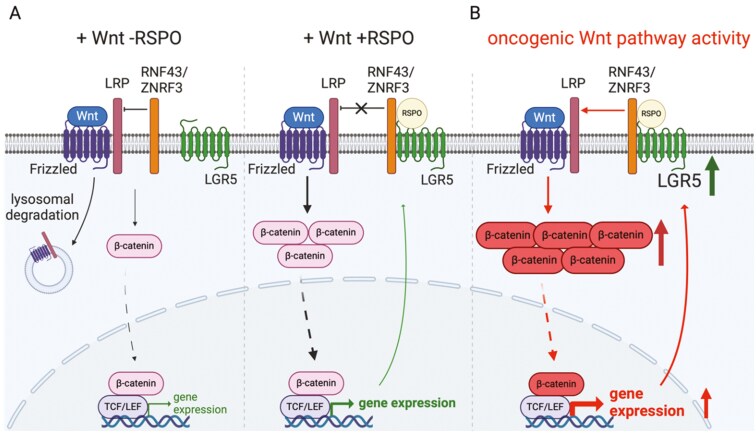
LGR5 potentiates Wnt pathway activity and acts in a feed forward loop. Wnt pathway activity is scaled to levels of Wnt ligands and Wnt receptors. (A) In the absence of RSPO ligand, the E3-ubiquitin ligases RNF43 and ZNRF3 earmark the Wnt receptors, Frizzled and LRP, for proteasomal degradation, thereby downregulating Wnt signalling. Upon binding of RSPO ligand to LGR5, LGR5 binds RNF43 and ZNRF3 and leads to the internalization and degradation of the complex. RSPO binding therefore uncouples negative regulation of Wnt pathway activity, potentiating the transcription of Wnt pathway target genes that include LGR5 itself. (B) Oncogenic mutations in the Wnt pathway lead to increased β-catenin levels, amplifying the Wnt transcriptional programme and increasing LGR5 expression.

The Wnt pathway is a cell–cell signalling pathway essential for coordinating metazoan development and regulating adult stem cell homeostasis [1]. The output of the Wnt pathway is a transcriptional programme that mediates its biological functions. LGR5 is a GPCR-like protein that potentiates Wnt pathway activity by increasing steady-state levels of its Frizzled (FZD) and LRP(5/6) receptors at the cell surface. The ubiquitin ligases RNF43 and ZNRF3 earmark FZD for internalisation and trafficking to the lysosome for destruction. R-spondin family members (RSPO1-4) are extracellular ligands for RNF43/ZNRF3 with LGR5 serving as the co-receptor [2, 3] (**Fig. 1A**). RSPO binding triggers RNF43/ZNRF43 auto-ubiquitination and internalization along with LGR5, and trafficking to the lysosome. The resulting increase in cell surface Wnt pathway receptors potentiates cellular responses to Wnt ligands (**Fig. 1A**). Interestingly, LGR5 is itself a target gene of the Wnt pathway, creating a positive feedback loop in stem cells that amplifies pathway activity (**Fig. 1A**). Lineage tracing experiments using a murine reporter of LGR5 expression have established LGR5 as stem marker in the small intestine and colon epithelia [4–6], gastric epithelial cells [5], hepatocytes [7, 8], and hair follicles [9], amongst others [10]. LGR5 expression also marks tumour-initiating cells (TICs) in intestinal epithelial tumour cells [11], forecasting similar expression in other tumour types including human TICs.

Oncogenic de-regulation of the Wnt pathway invariably leads to exaggerated stabilisation of β-catenin and precocious transactivation of target genes such as LGR5 (**Fig. 1B**). Increased LGR5 transcript levels have been reported for primary intestinal adenomas [11], gastric cancer [12], basal cell carcinoma [13], ovarian cancer [14], cervical cancer [15], glioblastoma [16], and precursor B-cell acute lymphoblastic leukaemia (pre-B ALL) [17, 18]. Most strikingly, LGR5 expression has been correlated with proliferation, migration, chemosensitivity, colony formation, and *in vivo* transplantation ability in colorectal cancer (CRC) (extensively reviewed in [19]). Furthermore, two landmark studies support a critical role for LGR5 expression in promoting metastatic growth in CRC: in mouse models and human tumour xenografts of CRC, LGR5 is not required for the metastatic spread but obligatory for the outgrowth of tumours at metastatic sites [20, 21].

It is thus no surprise that LGR5 has attracted a great deal of therapeutic interest owing to its overexpression in cancers with de-regulated Wnt pathway activity relative to low levels in healthy tissues, and the fact that tumour cells require LGR5 for proliferation. Furthermore, LGR5 localization on the plasma membrane makes it easily accessible for therapeutic targeting. However, the lack of suitable, well-validated, available antibodies has hampered both the detection of LGR5 expression in cancers and the development of LGR5-targeted therapies so far.

## α-LGR5—a versatile tool for patient stratification and developing LGR5-targeting immunotherapeutics

We have developed highly specific, high-affinity mouse monoclonal antibodies against the extracellular domain of human LGR5 (α-LGR5). All four clones that we successfully derived targeted a 15 amino acid epitope at the LGR5 N-terminus, and within their complimentary determining regions, differed by only 1-3 amino acids, explaining their similar binding affinities, with *Kd* values between 2-4 nM [22]. We proceeded with the highest affinity clone for humanization leading to an antibody with almost identical binding affinity for the epitope, *Kd* = 2 nM. Similarly, the corresponding scFv fragment retained high affinity binding to its epitope (*Kd* < 2 nM). α-LGR5 was extensively validated for a variety of research applications and detects cancer types with high levels of LGR5 (LGR5^+^ cells) - CRC (>80% of cases) and HCC (>90% of cases)—relative to healthy tissues that express low levels of the protein. We also detected highly elevated LGR5 transcript levels in pre-B ALL (>95% of cases). By and large, overexpression of LGR5 protein matched transcript data available in publicly accessible databases with one notable exception; while we observed a prominent increase in LGR5 protein levels in HCC, LGR5 transcript levels did not significantly vary from healthy liver tissues. For LGR5, we posit that protein levels are an accurate measure of ground truth expression and caution should be exercised when comparing bulk transcript expression levels.

A key application for monoclonal antibodies in immunotherapeutic development is their use as adaptable building blocks for development in a range of therapeutic modalities. This inherent versatility is apparent with clinically approved monoclonal antibodies targeting B cell markers such as CD19 and CD20, for which ADCs, bispecific T cell engagers, and CAR T cell therapies have been successful in treating B cell malignancies [23, 24] (**Table 1**). There are however limitations to the more widespread clinical use of monoclonal-based immunotherapeutics that include: (i) a lack of effective and safe new antigens that can be targeted in solid cancers; (ii) a lack of second-line antigens that can be targeted with patient relapse due to antigen escape [25], (iii) immunosuppression in the solid tumour microenvironment (TME) that attenuates the anti-tumour effector function of T cells and natural killer (NK) cells. α-LGR5-based immunotherapeutics could overcome these limitations owing to its high affinity and specificity for an important tumour antigen and its adaptability to multiple immunotherapeutic modalities.

**Table 1:** Clinically approved ADCs, bispecific T-ell engagers, and CAR T cells

Therapeutic modality	Target	First approval	Indication
**ADC**			
Adcetris (Brentuximab vedotin)	CD30	2011	Hodgkin lymphoma (HL) and anaplastic large cell lymphoma (ALCL)
Kadcyla (Trastuzumab emtansine)	HER2	2013	HER2^+^ breast cancer (BC)
Mylotarg (Gemtuzumab ozogamicin)	CD33	2017	Acute myeloid leukemia (AML)
Besponsa (Inotuzumab ozogamicin)	CD22	2017	B-cell precursor acute lymphoblastic leukemia (pre-B ALL)
Lumoxiti (Inotuzumab ozogamicin); withdrawn from market in 2021 (EU) and 2022 (U.S.)	CD22	2018	Hairy cell leukaemia
Polivy (Polatuzumab vedotin)	CD79b	2019	Diffuse large B-cell lymphoma (DLBCL)
Padcev (Enfortumab vedotin)	Nectin-4	2019	Urothelial cancer (UC)
Enhertu (Trastuzumab deruxtecan)	HER2	2019	HER2^+^ BC; HER2-low BC, gastric or gastroesophageal junction (GEJ) adenocarcinoma, non-small cell lung cancer (NSCLC); in 2024 approved for HER2^+^ solid tumours
Trodelvy (Sacituzumab govitecan)	Trop-2	2020	Triple-negative breast cancer (TNBC) and UC
Blenrep (Belantamab mafodotin); withdrawn from market in 2022 (U.S.) and in 2023 (EU)	BCMA	2020	Multiple myeloma (MM)
Zynlonta (Loncastuximab tesirine)	CD19	2021	DLBCL
Tivdak (Tisotumab vedotin)	Tissue factor	2021	Cervical cancer
Elahere (Mirvetuximab soravtansine)	FRa	2022	Epithelial ovarian, fallopian tube, or primary peritoneal cancer
Datroway (Datopotamab deruxtecan)	Trop-2	2025	HR^+^/HER2^–^ BC
**Bispecific T-cell engager**			
Blincyto (blinatumomab)	CD3/CD19	2014	pre-B ALL
Lunsumio (mosunetuzumab)	CD3/CD20	2022	follicular lymphoma
Kimmtrak (tebentafusp)	CD3/p100 peptide-HLA	2022	Uveal melanoma
Tecvayli (teclistamab)	CD3/BCMA	2022	MM
Columvi (glofitamab)	CD3/CD20	2023	DLBCL
Epkinly (epcoritamab)	CD3/CD20	2023	DLBCL, follicular lymphoma
**CAR T cells**			
KYMRIAH (tisagenlecleucel)	CD19	2017	Pre-B ALL, DLBCL, follicular lymphoma
TECARTUS (brexucabtagene autoleucel)	CD19	2020	Pre-B ALL, mantle cell lymphoma
ABECMA (idecabtagene vicleucel)	BCMA	2021	MM
BREYANZI (lisocabtagene maraleucel)	CD19	2021	DLBCL
YESCARTA (axicabtagene ciloleucel)	CD19	2022	DLBCL, follicular lymphoma
CARVYKTI (ciltacabtagene autoleucel)	BCMA	2022	MM

## α-LGR5 immunotherapeutic development

Three cancer types—CRC, HCC and pre-B ALL—emerge as prime indicator cancers for novel LGR5 immunotherapeutics. CRC is the third most commonly diagnosed cancer worldwide in 2022 [26] and in greater than 95% of cases, the Wnt signalling pathway is de-regulated through oncogenic mutations [27] with LGR5 playing a pivotal role in the survival of tumour cells and their metastatic proliferation (extensively reviewed in [19]). While immune checkpoint inhibition (ICI), such as α-PD1 and α-CTLA4 antibodies, have shown remarkable success in treating refractory mismatch-repair deficient, microsatellite unstable CRC which make up only 12-15% of all CRC cases [28, 29], for the majority of CRC patients, ICI has not yet delivered [30, 31]. Similarly, in HCC, which accounts for 1 in 10 cancer-related deaths in men and 1 in 20 in women [26], the Wnt/β-catenin signalling pathway is altered in 54% of all cases, primarily through activating mutations in *CTNNB1* (37%), encoding β-catenin and inactivating mutations in *AXIN1* (11%) [32]. These Wnt/β-catenin-driven tumours, particularly of the non-proliferative subtype, are characterized by immune exclusion and resistance to ICIs [33], highlighting the need for biomarkers to improve patient stratification and for novel therapies potentially overcoming immune exclusion. In pre-B ALL, a haematological malignancy, aberrant signalling pathways, including Wnt, drive the proliferation of immature B-cell precursors [23, 24]. While pre-B ALL predominantly affects children and adolescents under 20 years of age and has good outcomes upon therapy [23, 24], outcomes in relapsed/refractory cases or older patients are much poorer and tumour antigen escape is a recognized problem [34]. In the next sections, we discuss the advantages and disadvantages of the different α-LGR5 immunotherapeutic modalities that we have developed for targeting CRC, HCC, and pre-B-ALL.

## α-LGR5 antibody drug conjugates (ADCs)

**Figure 2.**
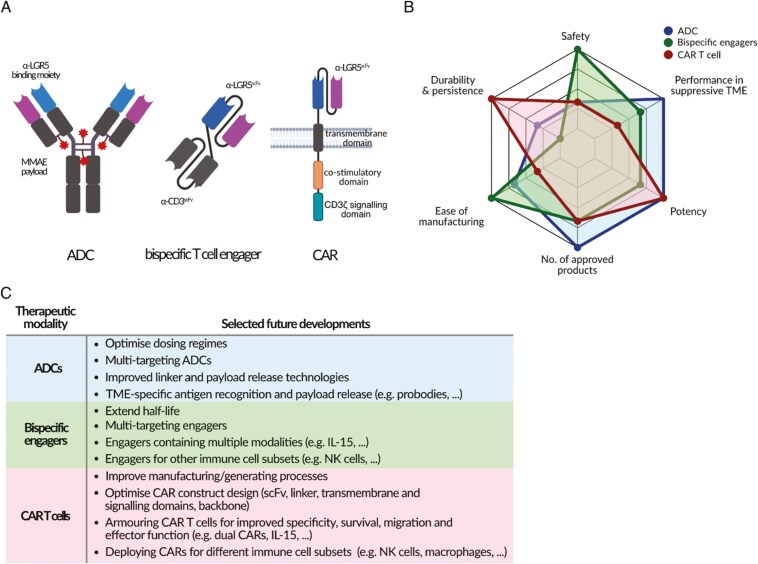
Strengths and weaknesses of ADC, bispecific, and CAR modalities. (A) Cartoon illustration of ADCs, bispecific T-cell engagers, and CARs for which α-LGR5 and α-LGR5^scFv^ have been used as building blocks. (B) Radar plot of different characteristics of ADC, bispecific engagers, and CAR T-cell immunotherapies. Each immunotherapeutic demonstrates a distinct profile of strengths and weaknesses. While ADCs are highly effective against both liquid and solid tumours, their efficacy is limited by off-target toxicity and PK/PD constraints. Bispecific engagers offer a cost-effective, off-the-shelf therapeutic option, but suffer from a short half-life and limited efficacy in immunosuppressive TMEs. CAR T cells provide highly specific tumour killing, bypass MHC restriction, and potentially achieve long-term persistence and tumour control. Despite these advantages, their widespread use is limited by high manufacturing costs, CRS, on-target/off-tumour toxicities, and poor migration into solid tumours. (C) Selected future developments in the ADC, bispecific engager and CAR T cell space addressing current limitations.

ADCs consist typically of an antibody conjugated to a cytotoxic payload through a linker (**Fig. 2A**). The underlying mechanism for all approved ADCs is the recognition of a cell surface antigen by the antibody moiety, internalization, payload release in the lysosome and tumour cell death. ADCs can also block oncogenic signalling pathways or induce antibody-dependent cellular cytotoxicity (ADCC) via their Fc region [35]. The field of ADC development is exploding, fuelled by antibody moieties targeting unique tumour associated antigens, and by technological innovations in ADC design. There are currently 14 ADCs with FDA approval composed of 11 unique antibody targets (**Table 1**). Furthermore, ADCs with more than 50 unique targets are in different phases of clinical development and of these, approximately 85% focus on solid tumour indications, emphasizing the broad clinical scope of ADCs [36].

Despite their enormous potential for targeting a wide range of cancer types, ADC treatment is prone to toxicity-related side effects that arise from the antibody targeting healthy tissues as well as the premature, off-target release of their cytotoxic payload (**Fig. 2B**), narrowing expected therapeutic windows [37]. Additionally, ADCs can form long-lasting albumin-linker adducts, unintentionally increasing their half-life and systemic exposure [38].

To address these challenges newer generations of ADCs incorporate structural innovations in their antibody, linker, and payload (reviewed in [39]; **Figure 2C**). These include bi-specific ADCs that simultaneously target two tumour antigens [40] and probody-drug conjugates, where the antigen-binding domain is only activated in the tumour microenvironment [41]. Advances in cleavable and non-cleavable linkers include variations in lengths and chemistry, enabling optimization of different drug-antibody ratios and the delivery of more than one payload class. Apart from potent chemotherapeutic agents, novel ADCs explore the potential of different payloads including immune-stimulating antibody conjugates [42] and proteolysis-targeting chimeras [43] (**Fig. 2C**).

The α-LGR5 that we have developed has excellent characteristics for serving as an effective ADC: the antibody has high affinity and specificity for LGR5^+^ cancer cells, is extremely rapidly internalized when bound to LGR5 (<5 minutes rate of internalization) and is transported to the lysosomal compartment [22, 44]. Importantly, a side-by-side comparison with trastuzumab (α-HER2)—an antibody successfully used as an ADC targeting lysosomal-mediated activation (**Table 1**)—indicated a greater than 40-fold decreased rate of internalization (>3 hours for 50% internalization) for trastuzumab compared to α-LGR5 [22]. The rapid internalization kinetics of LGR5 indicate enhanced safety with a reduced chance of premature payload release.

The α-LGR5 ADC was created by attaching the microtubule toxin monomethyl auristatin E (MMAE) using an ultra-stable sulfatase cleavable linker with an exact drug antibody ration of 4, developed by the Spring laboratory [45]. *In vitro* the α-LGR5 ADC exhibited potent cytotoxicity against pre-B ALL and CRC cell lines [22]. We have also tested the ability of α-LGR5 ADC to target CRC organoid models that we had stratified for LGR5 expression levels. Unsurprisingly, sensitivity to α-LGR5 ADC treatment increased proportionally to LGR5-expression indicating the potential for an accessible therapeutic window that can be exploited to eliminate LGR5^+^ tumour cells while avoiding on-target/off-tumour toxicity to healthy cells [22]. Furthermore, in the pre-B ALL NALM6 *in vivo* model, treatment with α-LGR5 ADC had tremendous efficacy, reducing tumour burden to less than 1% of control-treated animals [22]. Although we have confirmed an accessible therapeutic window for α-LGR5 ADC, further studies are required to assess toxicology for which we are designing a humanized LGR5 mouse model.

## LGR5-based bispecific engagers for T-cell recruitment to tumour cells

Bispecific T-cell engagers are engineered proteins consisting of an scFv fragment to CD3—a component of the T-cell receptor complex as well as an scFv to the B-cell marker CD19 connected via a linker (**Fig. 2A**). Bispecific T-cell engagers are the founding member of bispecific engagers that combine protein binding modules to immune cells and cell surface proteins expressed by cancer cells. The combination of a high-affinity, high-specificity targeting system with the inherent cytotoxic properties of T cells has led to their significant clinical successes against haematological malignancies and six bispecific T-cell engagers are currently clinically approved (**Table 1**).

Despite these successes, clinical application of bispecific T-cell engagers remains limited due to several challenges (**Fig. 2B**). One major issue is their short half-life, which necessitates prolonged and frequent intravenous (iv) infusions, e.g. blinatumomab has a half-life of only 2.1 hours [46]. Another challenge is the toxicity associated with bispecific therapies, including cytokine release syndrome (CRS) [47]. Although CRS in bispecific therapies is generally less severe than in CAR T-cell therapies, it still must be managed carefully [48]. Tumour relapse can occur due to antigen escape or cancer immunoediting, including PD-L1 upregulation [49, 50].

To overcome limitations of bispecific T-cell engagers, several innovations in the design of bispecific molecules are being explored in the clinic [51–53] (**Fig. 2C**). One approach has been to increase half-life by adding an Fc region from IgG antibodies or incorporating albumin-binding domains, thereby protecting the bispecific engagers from rapid degradation [54]. Bispecific engagers have also been designed with low-affinity CD3 binding to reduce excessive T-cell activation [55]. In addition, the engagement of different immune cells such as NK cells or γδ T cells is being explored [56] (**Figure 2C**). Other promising improvements involve enhancing potency and tumour cell specificity with multi-specific T-cell engagers to target multiple antigens or incorporating α-PD-L1 components to mitigate immune escape [1].

We created a novel bispecific T-cell engager by fusing the α-LGR5 scFv fragment to the α-CD3 scFv fragment from Blinatumomab to create the α-LGR5 bispecific [22]. The strategy was effective *in vitro* and co-culture of human peripheral blood mononuclear cells (PBMC) and LGR5^+^ NALM6 (pre-B ALL) tumour cells in the presence of α-LGR5 bispecific led not only to robust CD4^+^ and CD8^+^ T-cell activation but also potent and specific tumour cell killing [22]. Two treatments with 100 mg α-LGR5 bispecific and 7–10 million PBMCs led to a small yet significant 2-fold reduction in tumour load in a pre-B ALL model *in vivo* [22]. Immunohistochemical staining using α-LGR5 revealed that the residual tumour retained LGR5 expression, suggesting limitations in the half-life of the α-LGR5 bispecific or the treatment regimen. Our initial pilot experiments with α-LGR5 bispecific bode well for treatment of LGR5^+^ cancers. In future experiments, we plan to optimize the dosing regimen and time period, and as well, explore molecular designs to improve stability and efficacy, such as an α-LGR5 tri-specific that contains the IL-15 moiety for enhanced T cell function.

To evaluate the therapeutic window, we used LGR5^low^ and LGR5^high^ expressing pre-B ALL tumour cells and PDXs, as well as CRC cell lines to assess their susceptibility to CD8^+^ T cell-mediated killing in the presence of the α-LGR5 bispecific [22]. Experiments revealed that LGR5^high^ expressors were eliminated at significantly higher rates than their LGR5^low^ expressing counterparts. These *in vitro* results are encouraging and support the pursuit of further *in vivo* studies. In parallel, it might be advantageous to explore alternative α-LGR5 bispecific designs, for example, recruitment/activation of NK cells is likely to be less toxic than T cells owing to lack of CRS [57] (**Figure 2C**).

## Chimeric antigen receptors (CARs) utilizing α-LGR5^scFv^

The first CAR was designed by Eshhar *et al.* in 1989 [58]. CARs consist of an antigen-binding domain—most often an antibody-derived scFv-, a hinge domain, a transmembrane domain and the intracellular T-cell receptor (TCR) signalling domain CD3ζ as well as co-stimulatory domains. (**Fig. 2A**). T cells expressing a CAR can recognize tumour antigens independently from MHC presentation, bypassing one of the primary immune evasion strategies, which is the loss of MHC-associated antigen presentation by tumour cells [59]. Furthermore, as ‘living drugs’ CAR T cells exhibit intrinsic cytotoxic capacities and distinct pharmacokinetic behaviours [60] that can achieve long-term remission with a single infusion [61]. There are currently 6 clinically approved CAR T cell therapies that target 2 cancer antigens, CD19 and BCMA (**Table 1**).

Despite many successful CAR T-cell treatments in patients with B-cell malignancies, there are several intrinsic obstacles that have proven limiting. Perhaps the greatest roadblock to widespread clinical use of CAR-T cells is the incredibly poor performance in targeting solid tumour cancers. Although dozens of CAR T cell therapies are in early clinical trials for solid tumours, none have been clinically approved (reviewed in [62]). This is largely due to an inhospitable TME that biochemically attenuates T-cell effector function [63] and physically and biochemically suppresses migration to tumour sites [64] (**Fig. 2B**). Moreover, there is a sparsity of solid tumour antigens that are specifically overexpressed on cancer cells.

Treatment-associated toxicities, primarily severe CRS, are major concerns as are the lack of CAR T-cell persistence in patients owing to manufacturing protocols that enrich for terminally differentiated effector T cells with limited self-renewal capacity [65]. On-target/off-tumour toxicity is another common problem for CAR T cells: in the case of α-CD19, CAR T cells collaterally target healthy B cells leading to hypogammaglobulinemia. While the toxicity is easily manageable, on-target/off-tumour toxicities may prove more severe for targetable antigens in solid tumour cancers [66]. In addition, antigen escape with loss of CD19 expression has been observed in up to 25% of the patients treated with α-CD19 CAR T cells [67, 68].

In recent years many new improvements have been proposed to address the challenges underpinning CAR-T cell treatment. These include the generation of armoured CARs containing modified signalling domains or additional receptors, CAR T cells with different anti-tumour cargoes (extensively reviewed in [69]), as well as improved manufacturing protocols that enrich for long-lasting memory-like CAR T cells [70] (**Fig. 2B**).

We developed α-LGR5^scFv^ CAR T cells for targeting both solid tumour cancers such as CRC and HCC as well as LGR5^+^ B cell malignancies [22]. We hoped to tap into the prolonged effector function and persistence of CAR T cells that distinguishes them from ADC and bispecific engager strategies that rely on multiple administrations. We based the design of our α-LGR5^scFv^ CAR construct on the clinically approved 2nd generation CAR that incorporates either the CD28 or 4-1BB co-stimulatory domain, respectively. α-LGR5^scFv^ CAR T cells were generated by lentiviral transduction of CD8 T cells. The resultant α-LGR5^scFv^ CAR T cells exhibited potent and specific killing of LGR5-overexpressing cells as well as LGR5^+^ pre-B ALL, CRC, and HCC tumour cell lines [22].

In a pre-B ALL *in vivo* model we achieved a 3 to 5-fold reduction in overall tumour burden, with a significant decrease in residual tumour cells in the bone marrow niche [22]. While *in vivo* α-LGR5^scFv^ CAR T cells efficacy was apparent, we were unable to evaluate on-target/off-tumour toxicity in LGR5^+^ stem cell compartments—*in vivo* toxicology remains a challenge in murine models because α-LGR5 does not cross-react with the murine LGR5 protein. This is particularly relevant for evaluating the utility of α-LGR5^scFv^ CAR T cells that have a much longer half-life relative to molecular therapies such as ADCs and bispecific engagers. To evaluate a therapeutic window for α-LGR5^scFv^ CAR T cell treatment, we used LGR5^low^ and LGR5^high^ expressing pre-B ALL tumour cells and PDXs, as well as CRC cell lines, showing that LGR5^high^ expressors were preferentially eliminated [22]. There is a question mark over the utility of α-LGR5^scFv^ CAR T cell therapy for solid tumour cancers that will be assessed in future experiments using appropriate *in vivo* models.

Overall, α-LGR5 CAR T cells demonstrated strong efficacy in killing LGR5-expressing tumour cells *in vitro* and promising activity against pre-B-ALL tumours *in vivo*. Provided we obtain promising pre-clinical efficacy for α-LGR5^scFv^ CAR T cells in murine models of human CRC and HCC with a favourable toxicology profile, α-LGR5^scFv^ CAR T cell therapy could emerge as an excellent treatment option, offering the advantage of long-term persistence and unique tissue penetration that ADCs and bispecific engagers cannot achieve.

## Outlook

ADCs, bispecific T-cell engager and CAR T cells are promising immunotherapeutics with distinct modes of action, pharmacokinetic and toxicity profiles as well as production challenges. All three modalities have been approved for the treatment of B cell malignancies, and each brings unique advantages to benefit patients [71–73]. For example, bispecific engagers are cost-effective, off-the-shelf therapeutics that can be administered dose-dependently and carry a lower risk for CRS compared to CAR T cell therapy [74]. However, bispecific engagers are short-lived compared to CAR T cell therapies that have the potential for durable responses by generating long-lasting memory T-cell populations. These complementary strengths suggest a role for both therapies in B-ALL, where bispecific engagers seem to be more effective against minimal residual disease in B-ALL, whereas CAR T cells seem to have a superior event-free survival, particularly in those with r/r disease and high-disease burden [75].

A key advantage of targeting LGR5 is its unique overexpression in multiple, yet biologically distinct, Wnt pathway-dysregulated tumour types, including CRC, HCC, and pre-B ALL. Our own work on LGR5 emphasizes potential strengths of developing ADCs, bispecific engagers and CAR T cells against the same target in parallel. Each of our α-LGR5 immunotherapeutic modalities possesses distinct pharmacokinetic properties and mechanisms of action. We can exploit their complementary strengths, allowing for calibration of patient-specific target antigen expression in tumour and healthy tissues, the patient’s tumour load, metastasis profile, and their own immune system [76]. CAR T cells offer prolonged persistence and durable tumour control, but their efficacy is often limited by an immunosuppressive TME. In contrast, ADCs deliver potent cytotoxic payloads that act independently of immune cell infiltration, making them particularly promising for immune-excluded tumours. At the same time, bispecific engagers can rapidly recruit T cells to kill LGR5-expressing tumour cells, providing an off-the-shelf therapeutic with flexible dosing options.

With regard to the α-LGR5 ADC, we expect similar *in vivo* efficacy for *in vivo* models of CRC and other solid tumour types based on two previous studies that used a similar ADC format: one study observed eradication of CRC tumours initiated by the engraftment of LoVo cells over a 30-day treatment time course [44], while another, using the hu8E11v2 antibody demonstrated similar *in vivo* efficacy in targeting LoVo engrafted tumours [77]. hu8E11v2 was also used in a subsequent study in breast cancer and demonstrated reasonable efficacy in an *in vivo* tumour model. However, since these results, there have been no further academic or clinical studies of these two ADCs.

Importantly, we anticipate particularly robust responses for α-LGR5 ADC in certain cases of HCC. In our study we found that >80% of HCC cases overexpress the LGR5 protein [22]. Interestingly, the highest 30% of LGR5 expressors fall into the non-proliferative class of HCC, characterized by activating mutations in β-catenin (or rarely, AXIN1) that display an immune desert phenotype [33, 78]. We believe that an α-LGR5 ADC will be particularly effective in this subclass where immune cell-based therapies are likely to be excluded or inactivated.

Additionally, the development of α-LGR5-based CAR T cells holds promise for CRC, HCC, and haematological malignancies such as pre-B ALL. Currently, CNA3103, an α-LGR5^scFv^-based CAR T cell therapy, is in Phase 1/2a clinical trials for the indicator metastatic CRC (NCT05759728). While α-LGR5^scFv^ CAR T cell treatments may be efficacious for solid tumour cancers, B-ALL may also prove to be an appropriate disease target. CD19 antigen loss is a major reason for therapy failure in B-ALL, despite the availability of potent therapies like α-CD19 CAR T cells [79]. Therefore, α-LGR5^scFv^ CAR T cells may serve as a salvage therapy for refractory, CD19^–^ B-ALL.

Taken together, interactions between the immune system and cancer are highly complex and require novel and adaptable antibodies such as α-LGR5 to provide opportunities for patient stratification as well as effective immunotherapeutics for improved clinical outcomes.

## Data Availability

Not applicable.
